# Author Correction: The influence of anxiety and fear of COVID-19 on vaccination hesitancy among postsecondary students

**DOI:** 10.1038/s41598-022-27000-5

**Published:** 2023-01-06

**Authors:** Andrej Šorgo, Nuša Crnkovič, Katarina Cesar, Špela Selak, Mitja Vrdelja, Branko Gabrovec

**Affiliations:** 1 Faculty of Natural Science and Mathematics, University of Maribor, Koroška Cesta 160, 2000 Maribor, Slovenia; 2 National Institute of Public Health, Trubarjeva 2, 1000 Ljubljana, Slovenia

Correction to: *Scientific Reports* 10.1038/s41598-022-25221-2, published online 29 November 2022

The original version of this Article contained an error in the Funding section.

“This publication arises from the “Measures in the Field of COVID-19 Spread Management with a Focus on Vulnerable Populations” project, funded by Slovenia and the European Social Fund, of the European Structural and Investment Funds, covered by Regulation (EU) No. C2711-21-053701 of the European Parliament and the Council (the Common Provisions Regulation).”

now reads:

“The study was conducted in Slovenia as part of a NIJZ project. The project title is “Measures to manage the spread of COVID-19 with a focus on vulnerable groups of population”. This research is co-financed by the Republic of Slovenia and the European Union under the European Social Fund in the framework of the EU response to the COVID-19 pandemic (Grant No. C2711-20-054101). The content of this article represents the views of the authors only and is their sole responsibility; it cannot be considered to reflect the views of the European Commission or any other body of the European Union. The European Commission does not accept any responsibility for the use that may be made of the information it contains.”

The original Article has been corrected.

